# Supplementary material for: Machine Learning Methods for Gene Selection in Uveal Melanoma
Source: Int J Mol Sci. 2024 Feb 1;25(3):1796. doi: 10.3390/ijms25031796 (PMC10855534; doi:10.3390/ijms25031796)
Supplement: Supplementary file 1 [file ijms-25-01796-s001.zip › ijms-2820766-supplementary.pdf]

Supplementary figures

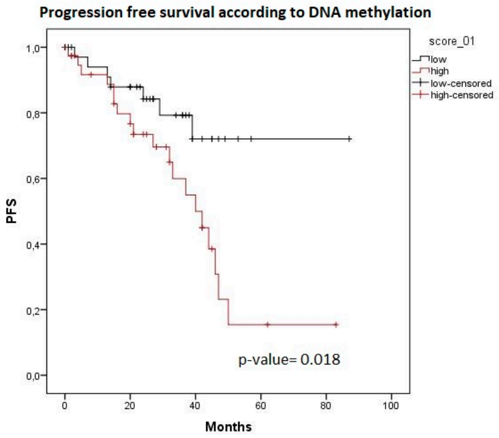

Figure S1. KM curves of patients with a high or low MGS considering the differentially methylated probes detected by SAM analysis on the two jSVD related clusters. Low score curve is in black, while the high score curve is reported in red.

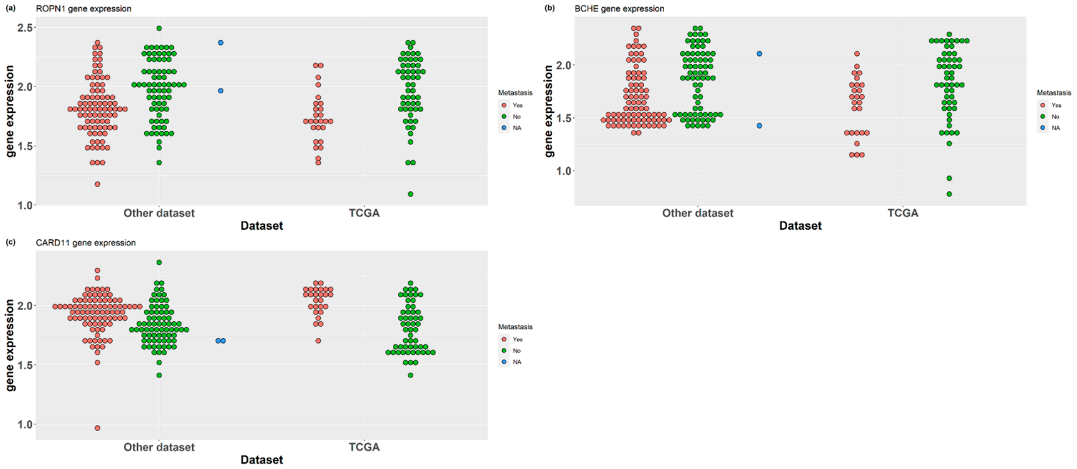

Figure S2. Expression level and Metastasis state in patients of the Piaggio et al 2022 dataset used for computing the MGS score. Patients from the TCGA dataset and other datasets are shown separately.

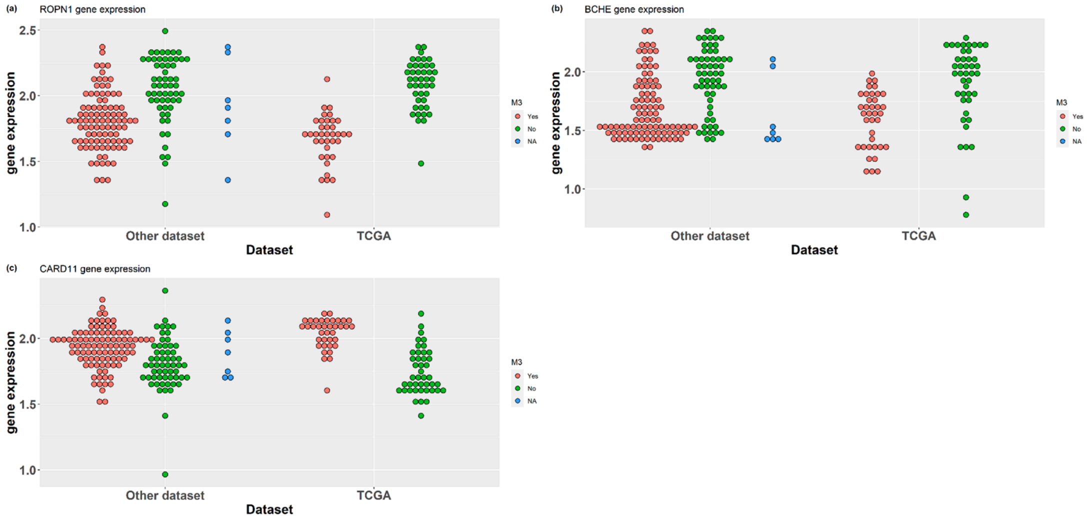

Figure S3. Expression level and M3 state in patients of the Piaggio et al 2022 dataset used for computing the MGS score. Patients are divided as members of the TCGA dataset and all other samples.
